# Preoperative prevalence and risk factors for thromboembolic events following tibial plateau fractures: a retrospective analysis of an underestimated risk

**DOI:** 10.1007/s00068-026-03371-6

**Published:** 2026-10-21

**Authors:** Johannes Herold, Emilie Dagmar Lorenz, Hannah Eymann, Felix Lohse, Konrad Kamin, Eric Jörg Walther, Marcel Mäder, Ivonne Panchyrz, Sebastian Werth, Norbert Weiss, Christian Kleber, Klaus-Dieter Schaser

**Affiliations:** 1 University Center for Orthopedics, Trauma and Plastic Surgery, University Hospital Carl Gustav Carus, Fetscherstraße 74, 01307 TU Dresden, Germany; 2 Department of Orthopedic, Trauma and Plastic Surgery, Leipzig University Medical Center, Leipzig, Germany; 3 University Center for Vascular Medicine & Department of Medicine III -Section Angiology, University Hospital Carl Gustav Carus, TU Dresden, Germany; 4 Center for Evidence-Based Healthcare, University Hospital Carl Gustav Carus, TU Dresden, Germany

**Keywords:** Tibial plateau fracture, Venous thromboembolism, Duplex ultrasound screening, External fixation, Asymptomatic deep vein thrombosis, Injury severity

## Abstract

**Background:**

Tibial plateau fractures (TPF) are complex intra-articular injuries carrying a high risk of deep vein thrombosis (DVT), muscular vein thrombosis (MVT) and pulmonary embolism (PE). Preoperative thrombosis is frequently clinically silent yet may cause serious complications, whereas its detection delays definitive surgery. This study assessed the prevalence and risk factors of preoperative venous thromboembolic events (VTE) in surgically treated TPF before and after implementation of routine duplex ultrasound screening.

**Methods:**

In this retrospective single-centre study at a level 1 trauma centre, all adult patients surgically treated for traumatic TPF (AO 41B/C) between January 2015 and August 2025 were reviewed (547 patients, 554 fractures). Until August 2020 duplex ultrasound (DUS) was performed only on clinical suspicion; from August 2020 onward routine preoperative DUS was progressively implemented. VTE were confirmed by DUS or CT angiography. Prevalences are reported per fracture and patient characteristics per patient; regression analyses were performed at fracture level, except for the patient-related model.

**Results:**

With DUS restricted to clinical suspicion (cohort A; 246 fractures), the detected prevalence of preoperative VTE was 3.7% (95% CI 1.9–6.8) and that of DVT 2.4% (1.1–5.2). Under routine screening (cohort B; 205 screened fractures) it was 20.0% (15.1–26.0) and 17.1% (12.5–22.8), respectively, 82.9% of DVT being distal. Given the different diagnostic strategies, this reflects the higher diagnostic yield of screening rather than a true increase in VTE. In cohort B, 28 of 41 VTE (68.3%) and 24 of 35 DVT (68.6%) were clinically silent. Two patients had in-hospital cardiac arrest from PE: one died from a DVT undetected before screening was introduced, the other had an asymptomatic DVT identified by screening. VTE was independently associated with pre-existing therapeutic anticoagulation (adjusted OR 2.96, 95% CI 1.10–7.98) and with the duration of external fixation (adjusted OR 1.11 per day, 1.02–1.20). High-grade closed soft-tissue injury (adjusted OR 2.41, 1.07–5.45), polytrauma (adjusted OR 2.24, 1.07–4.68) and initial external fixation (univariable OR 3.60, 1.76–7.35) were also associated with VTE, but none remained significant after accounting for fixation duration (adjusted OR 1.42, 0.52–3.89; 1.96, 0.76–5.05; 0.62, 0.12–3.27), i.e. risk was driven by time in the fixator rather than by external fixation as such.

**Conclusions:**

Surgically treated TPF carry a substantial burden of preoperative VTE, and routine DUS reveals a high proportion of clinically silent thrombosis that would otherwise remain undetected. Severe closed soft-tissue injury, pre-existing therapeutic anticoagulation and, above all, the duration of external fixation may help to identify patients at increased risk. As two diagnostic rather than two treatment strategies were compared, the study cannot determine whether screening improves outcomes or reduces PE; prospective evaluation of structured screening and risk-adapted thromboprophylaxis is warranted.

## Introduction

Among all intraarticular fractures, tibial plateau fractures (TPF) represent one of the most challenging entities, owing to their complex fracture morphology, frequent association with ligamentous and intraarticular injuries (osteochondral, meniscal), significant soft tissue damage, and the risk of both acute as well as late complications [1]. TPFs range from high-energy/high velocity injuries in active younger patients to low-energetic osteoporotic fractures in geriatric patients. They represent approximately 1% of total fractures, suggesting a relatively rare but increasingly prevalent injury as demonstrated by the rapidly increasing incidence rates [2, 3]. One contributing factor is the demographic change with an progressively aging but active population. These geriatric TPF are typically accompanied by a rise of complex multifragmentary fracture patterns resulting from decreased bone quality [4]. Moreover, essential ligamentous and intraarticular structures can also be injured. Soft tissue trauma including compartment syndrome is often observed and an advanced knee osteoarthritis may be preexisting. These aggravating factors independently or in combination inevitably complicate anatomic reduction and stable fixation, which makes TPF treatment very challenging and a task for experienced reconstructive surgeons.

Late complications following TPF include non-union, joint instability, stiffness and/or posttraumatic osteoarthritis, frequently necessitating revision surgery or subsequent conversion to total knee arthroplasty (TKA). Typical acute complications comprise neurovascular injury, fracture-related infection, and the development of deep vein thrombosis (DVT) or muscular venous thrombosis (MVT), which may progress to pulmonary embolism (PE). The overall occurrence of DVT is expected to rise, driven by the steadily growing prevalence of multiple comorbidities in older patients, which in turn are known to markedly increase the risk of cardiovascular and thromboembolic complications [5].

Pulmonary thromboembolism after DVT is among the most fearful and fatal complication associated with TPF. In addition, the preoperative diagnosis of thromboembolic events (VTE), especially the occurrence of preoperative DVT, regularly leads to a significant delay in the definitive surgical treatment, making articular reduction with anatomic reconstruction and fixation much more complex and difficult. Apart from complicated anatomic reconstruction with worse functional outcome and prolonged hospitalization and costs, post thrombotic syndrome with chronic pain; secondary varicosis and ulcers may be other adverse sequelae [6].

The trauma-induced hypercoagulable state, fracture dislocation with vascular traction injury and a systemic inflammatory response are known causative factors contributing to increased susceptibility for venous thrombosis. This risk is once more increased by prolonged pre- and postoperative immobilization, partial weight bearing and pain-related impairment of the muscle pump [7–12].

In the absence of thromboprophylaxis, the incidence of DVT in patients with TPF is up to 77% [13], while data reporting rates of asymptomatic DVT are sparse but seem to range up to 42% [14] and even 58% [10]. However, despite sufficient medical thromboprophylaxis, the incidence of DVT ranges from approximately 1.7% [15] to 26.7% in patients with TPF, with approximately 4.5% [16] of those being proximal, i.e. the DVT is localized in the popliteal vein or proximally.

The occurrence of DVT in patients with isolated fractures of the lower extremity has been described in numerous studies [17–22]. Although, previous studies, already cited in the guideline recommendation on the prophylaxis of venous thromboembolism, explicitly emphasized the enhanced risk of tibial plateau fractures [14, 23], most studies on MVT, DVT and PE focus on hip and knee arthroplasty or hip fractures. However, most previous studies on patients with TPF, who routinely received thromboprophylaxis, are either heterogeneous in design or lack details and evidence regarding identifying risk factors associated with MVT, DVT and PE [24–29]. As DVTs and MVTs following TPF may develop already preoperatively without any symptoms (silent/inapparent) in a high number of unidentified cases, establishment of guide-/timelines for monitoring and intervention [24] is not only very challenging but of equal relevance and importance.

This study aimed to analyze the incidence and risk factors of preoperative thromboembolic events, defined as deep vein thrombosis (DVT), muscular vein thrombosis (MVT), and pulmonary embolism (PE), in surgically treated tibial plateau fractures (TPF) in a cohort of 547 consecutive patients.

## Methods

### Inclusion and exclusion criteria

All patients treated for a traumatic tibial plateau fracture (TPF) at our level 1 trauma centre between January 2015 and August 2025 were identified retrospectively by a structured query of the institutional database using the ICD-10 diagnosis code S82.18 (*n* = 838). Inclusion criteria were an age of at least 18 years and surgical treatment of a TPF classified as AO type 41B or 41 C.

Fractures were excluded if they were classified as AO type 41 A, treated conservatively, or represented periprosthetic fractures following total knee arthroplasty, pathological fractures or isolated ligamentous avulsions, as were patients younger than 18 years (*n* = 291). This left *n* = 554 TPFs in *n* = 547 patients, including seven bilateral cases.

**Fig. 1:**
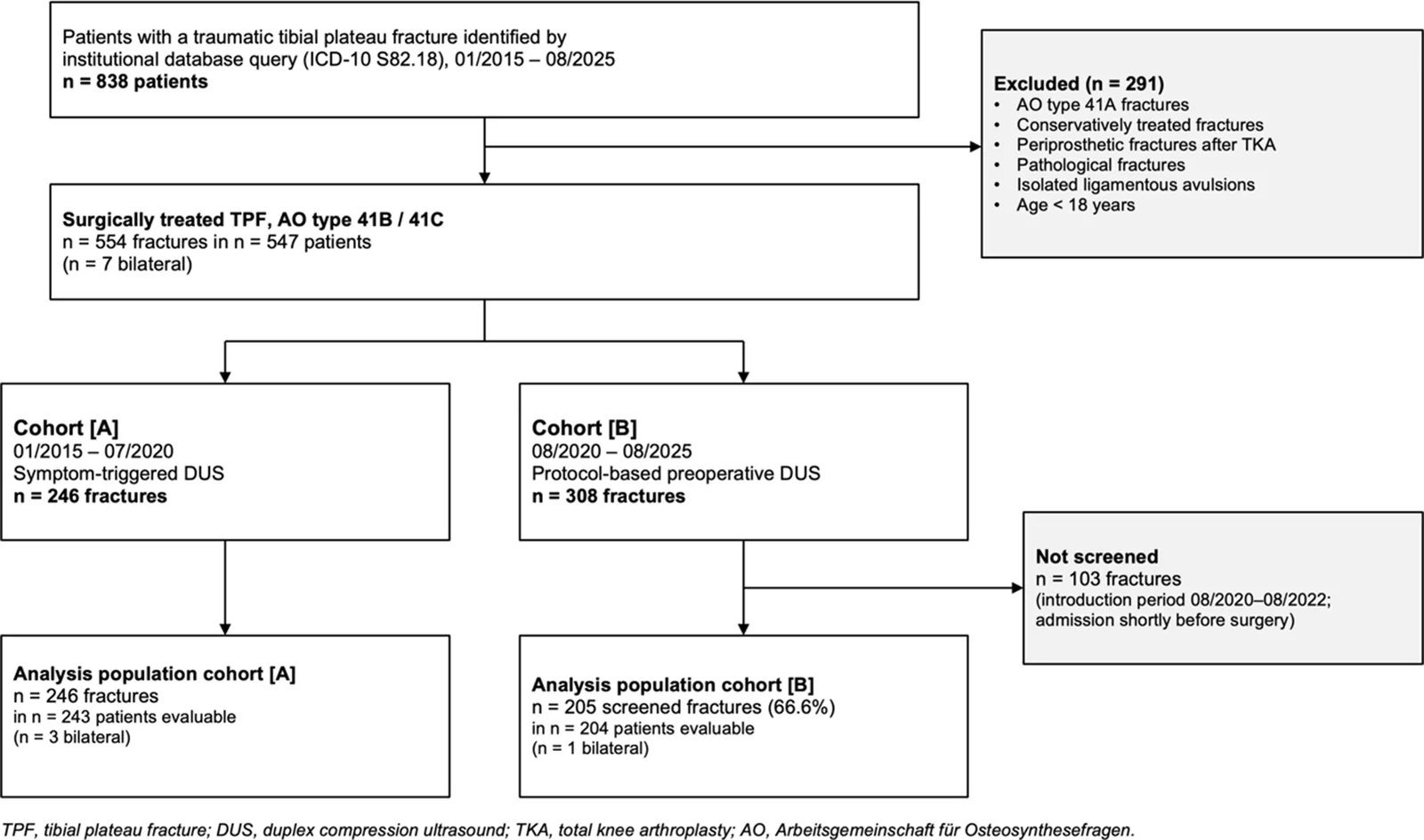
Flow of patients and fractures through the study, showing eligible and excluded fractures, fractures with and without preoperative duplex ultrasound screening, and the numbers entering each analysis. TPF, tibial plateau fracture; VTE, venous thromboembolic event; TKA, total knee arthroplasty

No further exclusion criteria were applied: in particular, all surgically treated TPFs were included irrespective of injury severity, injury mechanism or the presence of concomitant injuries, and patients with polytrauma or multiple injuries to the extremities were not excluded. The flow of all eligible, excluded, screened and non-screened fractures, and the numbers entering each analysis, is shown in Fig. 1. Ethical approval was obtained under the reference number BO-EK-289,062,023.

### DVT/MVT screening

Between 01/2015 and 07/2020, compression ultrasound to diagnose or rule out DVT/MVT was performed only in the case of clinical suspicion or manifest pulmonary embolism; this period comprises *n* = 246 TPFs (*n* = 243 patients, *n* = 3 bilateral) and is referred to as cohort [A]. In 2020 the management was changed following a lethal thromboembolic complication, and preoperative compression ultrasound was introduced as a routine component of the preoperative work-up.

Implementation was progressive and took approximately two years: during the introduction period (08/2020–08/2022) the protocol was applied inconsistently, whereas from 09/2022 onward preoperative DUS was performed reliably in every patient scheduled for surgical treatment of a TPF. Cohort [B] therefore comprises all *n* = 308 TPFs (*n* = 304 patients, *n* = 4 bilateral) treated between 08/2020 and 08/2025, of which *n* = 205 TPFs (*n* = 204 patients, *n* = 1 bilateral) actually underwent preoperative DUS and constitute the screened cohort analysed here. The *n* = 103 TPFs without preoperative DUS originate predominantly from the introduction period 08/2020–08/2022, during which the protocol was not yet applied consistently. After screening had become reliable in 09/2022, the remaining exceptions were patients who had initially been managed on an outpatient basis and were admitted only shortly before surgery, so that a preoperative DUS could no longer be scheduled in time. Missing examinations were not imputed; all prevalence estimates for cohort [B] refer exclusively to the 205 screened fractures, and the resulting potential for selection bias is addressed in the Limitations. Duplex compression ultrasound (DUS) was performed from the groin down to the distal calf to visualize all deep leg veins and the muscle veins according to established protocols. Isolated caval or iliac vein thrombosis was ruled out by demonstrating flow variation during the respiratory cycle [30–33].

Patients were subsequently stratified into two groups:

[A].  Patients before implementation of preoperative DVT/MVT screening, the recorded number of thrombosis corresponds to the prevalence of symptomatic thrombosis (MVT/DVT and PE).
[B]. Patients with preoperative DVT/MVT screening, the rate of recorded thrombosis reflects the total sum of symptomatic and inapparent/silent MVT, DVT and PE.

A venous thromboembolic event (VTE) was defined as any imaging-confirmed thrombosis of the deep leg veins (DVT), of the calf muscle veins (MVT), or a pulmonary embolism (PE) diagnosed before definitive surgical fixation. DVT was defined as a non-compressible deep vein segment on duplex compression ultrasound; proximal DVT as thrombosis involving the popliteal vein or any vein proximal to it, and distal DVT as thrombosis confined to the deep veins below the knee joint. MVT was defined as isolated thrombosis of the gastrocnemius and/or soleal veins without involvement of the axial deep veins and was recorded separately from DVT throughout. PE was confirmed by computed tomography pulmonary angiography.

A VTE was classified as symptomatic when imaging had been triggered by clinical signs or symptoms suggestive of thrombosis or pulmonary embolism and the diagnosis was subsequently confirmed, and as asymptomatic when it was detected by protocol-driven screening in a patient in whom no thrombosis-related sign or symptom had been documented.

Because swelling and pain are intrinsic to the fracture itself, this classification was based on the documented clinical indication for the examination rather than on the presence of limb symptoms alone. The Duplex ultrasound was performed by an angiologist.

The fracture, i.e. the injured limb, was the unit of analysis for all injury- and procedure-related variables, all prevalence estimates and all regression analyses, because both the exposures and the outcome are limb-specific; patient characteristics are reported at patient level. Seven patients sustained bilateral TPF (cohort [A] *n* = 3; cohort [B] *n* = 4, of whom *n* = 1 was screened) and contributed two fractures each, while their patient-related characteristics were counted once. Given that bilateral cases represent 1.3% of fractures, no correction for within-patient clustering was applied. Missing values were not imputed; all analyses were performed on the complete cases available for the respective variable.

### Anticoagulation algorithm

All patients with TPF at our center received basic medical thromboprophylaxis with low molecular weight heparin (LMWH), provided that no heparin-induced thrombocytopenia (HIT) was known. The standard substances used were Enoxaparin (4000 IU) or Certoparin (3000 IU) in their recommended dosage for high-risk DVT prophylaxis, once daily by subcutaneous injection. Dose adjustments of LMWH were implemented in patients with impaired kidney function and when oral anticoagulation or marked obesity (over 120 kg) was already present preoperatively. Medical DVT-prophylaxis was continued during the length of hospital stay and until full weight-bearing was achieved. In addition, mechanical thromboprophylaxis was performed using pneumatic plantar venous plexus compression (A-V impulse system Vadoplex, OPED), applied for 4 to 15 h daily depending on patient tolerance, with a cycle duration of 20 s, an inflation pressure of 130 mmHg, and an impulse duration of 1 s.

For patients with proximal DVT and PE who had contraindications for therapeutic anticoagulation or progression of the proximal DVT and PE during anticoagulation therapy the implantation of a vena-cava filter was performed. Patients who were already receiving oral anticoagulation on admission were managed according to the institutional perioperative protocol. The oral anticoagulant (phenprocoumon or a direct oral anticoagulant) was discontinued and substituted by low molecular weight heparin in an intermediate, i.e. half-therapeutic, dose, administered once daily in a weight-adapted dosage until definitive surgical treatment; the last preoperative dose was given on the evening before surgery. In these patients the prophylactic LMWH regimen described above was not applied in addition.

Antiplatelet monotherapy with acetylsalicylic acid was continued throughout the perioperative period unless it had been prescribed for primary prophylaxis only, in which case it was discontinued. Postoperatively, anticoagulation was resumed on the day of surgery in the same intermediate, half-therapeutic dose and was subsequently escalated to a full therapeutic dose as rapidly as the bleeding situation allowed, at the latest within 48 h.

**Fig. 2:**
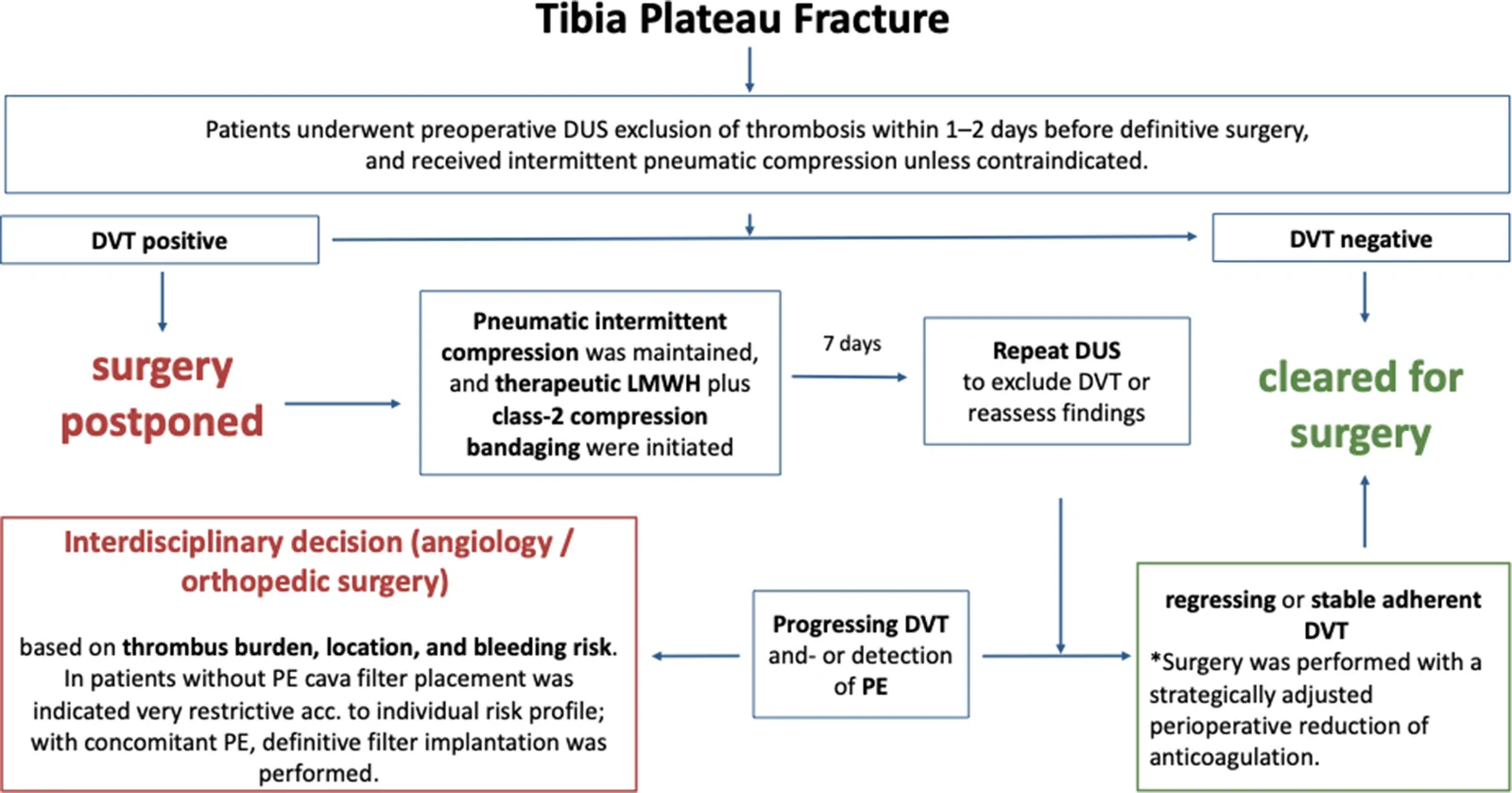
DVT screening algorithm for the preoperative management of TPF

If DVT could be definitively excluded preoperatively, surgical intervention was conducted as initially scheduled without any further postponements. For patients diagnosed with MVT, anticoagulation was increased to subtherapeutic levels and surgery still carried out under precaution. In cases of confirmed DVT, surgery was postponed, therapeutic anticoagulation was initiated, and class 2 compression bandaging was applied whenever clinically feasible. Patients were re-evaluated after 7 days. Those with stable or regressing thrombus burden were cleared for surgery with perioperative dose reduction of anticoagulation. In patients with progressing DVT or PE, further management was determined on an individual basis following interdisciplinary discussion between angiology and orthopedic surgery. The algorithm is shown in Fig. 2.

### Statistical analysis

Data plausibility was verified prior to analysis. Statistical analyses were performed using SPSS^®^ version 31.0 (IBM Corp., Armonk, NY, USA). Injury- and procedure-specific variables were analyzed on a per-fracture basis (*n* = 554), while patient characteristics were reported at the patient level (*n* = 547). Continuous variables are presented as means ± standard deviations (SD), and group differences were tested using the Mann-Whitney U test. Nominal and categorical variables were compared using chi-square or Fisher’s exact test, as appropriate. Statistical significance was set at *p* < 0.05. Prevalence estimates are reported with 95% confidence intervals (CI) calculated by the Wilson score method; differences between proportions are reported with 95% CIs according to the Newcombe hybrid score method. Missing values were not imputed. Descriptive analyses were based on the documented cases, and the number of documented cases is reported per variable in Table 1 wherever documentation was incomplete. The regression models were fitted on complete cases; variables with more than 10% missing data were reported descriptively only and were not entered into the multivariable models, in order to preserve the analytic sample and the events-per-variable ratio. Postoperative haemoglobin met this univariable screening threshold (*p* < 0.2) but was not entered into the multivariable models: as a postoperative measurement it cannot represent a preoperative risk factor for VTE, and its inclusion would introduce reverse-causation bias.

Multivariable logistic regression was performed to identify factors independently associated with VTE (MVT + DVT + PE) in the screened cohort [B]. All univariable and multivariable analyses were performed at fracture level. Variables with *p* < 0.2 in univariable analysis were considered as candidates. Because only 41 events were available, the covariates were not entered into a single model but grouped into four separate, clinically defined models: (i) age, body weight and pre-existing antithrombotic therapy; (ii) grade of closed soft-tissue injury, AO classification, injury pattern, open fracture and injured side; (iii) use and duration of external fixation; and (iv) grade of closed soft-tissue injury, injury pattern and duration of external fixation. With 41 events these correspond to events-per-variable ratios of 13.7, 8.2, 20.5 and 13.7. Odds ratios are reported per unit increase for continuous variables and against the stated reference category for categorical variables. Collinearity was assessed by the Spearman correlation matrix and variance inflation factors, and model fit by the Hosmer–Lemeshow test, Nagelkerke’s R² and the c-statistic.

## Results

### Incidence of thrombotic events

In this retrospective study the medical records of all patients were reviewed in full, especially regarding potential symptoms and manifestation of thrombotic events.

In cohort [A] with 246 TPFs (*n* = 243 patients; *n* = 3 bilateral), symptomatic preoperative DVT was confirmed in 6 TPFs, including 1 with concomitant PE. Additionally, 2 patients presented with PE in the absence of detectable DVT, and *n* = 1 patient exhibited symptomatic MVT, yielding an overall detected prevalence of venous thromboembolic events (VTE) of 3.7% (9/246; 95% CI 1.9–6.8). The detected prevalence of DVT alone (*n* = 6, including the patient with concomitant PE) was 2.4% (95% CI 1.1–5.2).

As imaging was performed only on clinical suspicion during this period, these figures represent the prevalence of clinically apparent (symptomatic) VTE and do not capture clinically silent events.

In cohort [B], among 308 TPFs (*n* = 304 patients; *n* = 4 bilateral) treated since 08/2020, 205 TPFs (*n* = 204 patients; *n* = 1 bilateral) underwent preoperative DUS, corresponding to a screening coverage of 66.6% (95% CI 61.1–71.6). In these screened fractures the detected prevalence of DVT was 17.1% (35/205; 95% CI 12.5–22.8). Of these 35 DVT, 24 (68.6%) were clinically silent and 11 (31.4%) symptomatic, the latter including one patient with concomitant PE. MVT was detected in 6 fractures (2.9%; 95% CI 1.3–6.2), of which 4 were clinically silent and 2 symptomatic. The overall detected prevalence of VTE was 20.0% (41/205; 95% CI 15.1–26.0). Taken together, 28 of 41 events (68.3%) were clinically silent, corresponding to 13.7% (95% CI 9.6–19.0) of all screened fractures, whereas 13 events (31.7%), corresponding to 6.3% (95% CI 3.7–10.5), were symptomatic and would also have been detected without a screening protocol.

Because the two cohorts were investigated with fundamentally different diagnostic strategies, the difference between them has to be interpreted as a difference in diagnostic yield rather than as a change in the true occurrence of VTE. The detected VTE prevalence was 16.3% points higher under routine screening than under symptom-triggered imaging (20.0% vs. 3.7%; 95% CI for the difference 10.5–22.6; *p* < 0.001), and the detected DVT prevalence was 14.6% points higher (17.1% vs. 2.4%; 95% CI 9.3–20.5; *p* < 0.001). Almost the entire difference was accounted for by clinically silent events, whereas the prevalence of symptomatic VTE did not differ significantly between the cohorts (6.3% vs. 3.7%; difference 2.7% points, 95% CI − 1.4 to 7.2; *p* = 0.197).

**Fig. 3:**
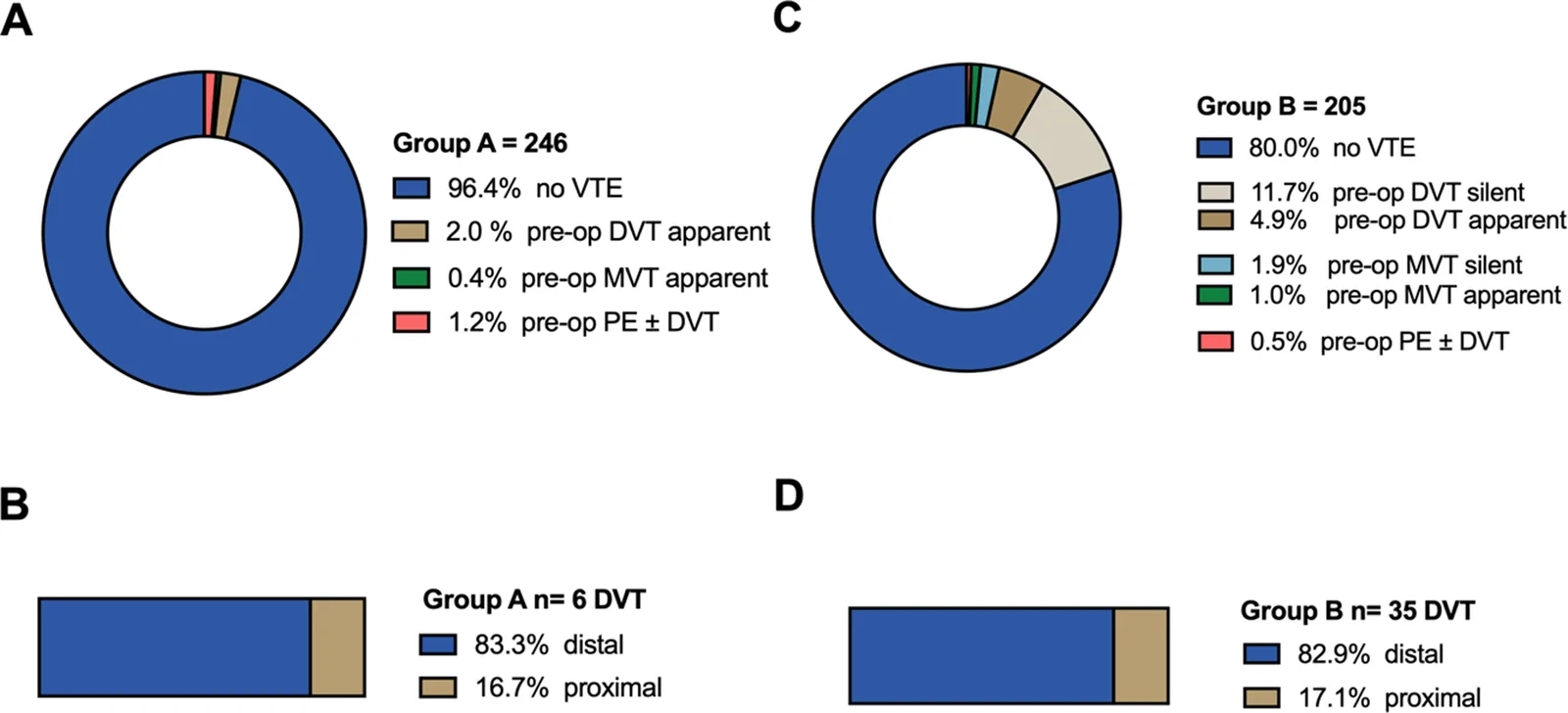
[**A**] percentage distribution of pre-operative VTE in TPFs in unscreened patients; [**B**] percentage distribution of proximal and distal DVT in the unscreened cohort; [**C**] percentage distribution of pre-operative VTE in TPFs in DUS screened patients; [**D**] percentage distribution of proximal and distal DVT in the screened cohort

Preoperative PE was detected in 3 of 246 fractures in cohort [A] (1.2%, 95% CI 0.4–3.5) and in 1 of 205 screened fractures in cohort [B] (0.5%, 95% CI 0.1–2.7); this difference was not statistically significant (*p* = 0.63), and the present design does not permit any conclusion as to whether screening reduces the occurrence of PE. Regarding thrombus location, 16.7% (1/6) of DVT in cohort [A] and 17.1% (6/35) in cohort [B] were proximal (popliteal, femoral or iliac veins), the remainder distal. For a detailed breakdown of all event categories, see Fig. 3.

Two patients experienced in-hospital cardiac arrest resulting from pulmonary embolism, one of whom died. In the deceased patient, the preoperative DVT had not been recognized prior to surgery, as systematic DUS screening had not yet been implemented, and the event represented a first-time thrombotic episode. The second patient had an asymptomatic, proximal DVT that was detected through preoperative DUS screening, and therapeutic anticoagulation was initiated accordingly. Control DUS demonstrated a stable condition. Following interdisciplinary consultation between angiology and orthopedic Surgery, definitive surgery was performed under appropriate precautionary measures.

Intraoperatively, the patient suffered cardiac arrest, requiring immediate extracorporeal cardiopulmonary resuscitation (eCPR), lysis, and interventional thrombectomy. The patient survived without neurological deficit and with good functional knee outcome.

Poly- or multiple trauma was documented in 79 of 243 evaluable patients (32.5%) in cohort [A] and in 69 of 204 evaluable patients (33.8%) in the screened cohort [B] (Table 1).

### Risk factors for the development of VTE

**Fig. 4:**
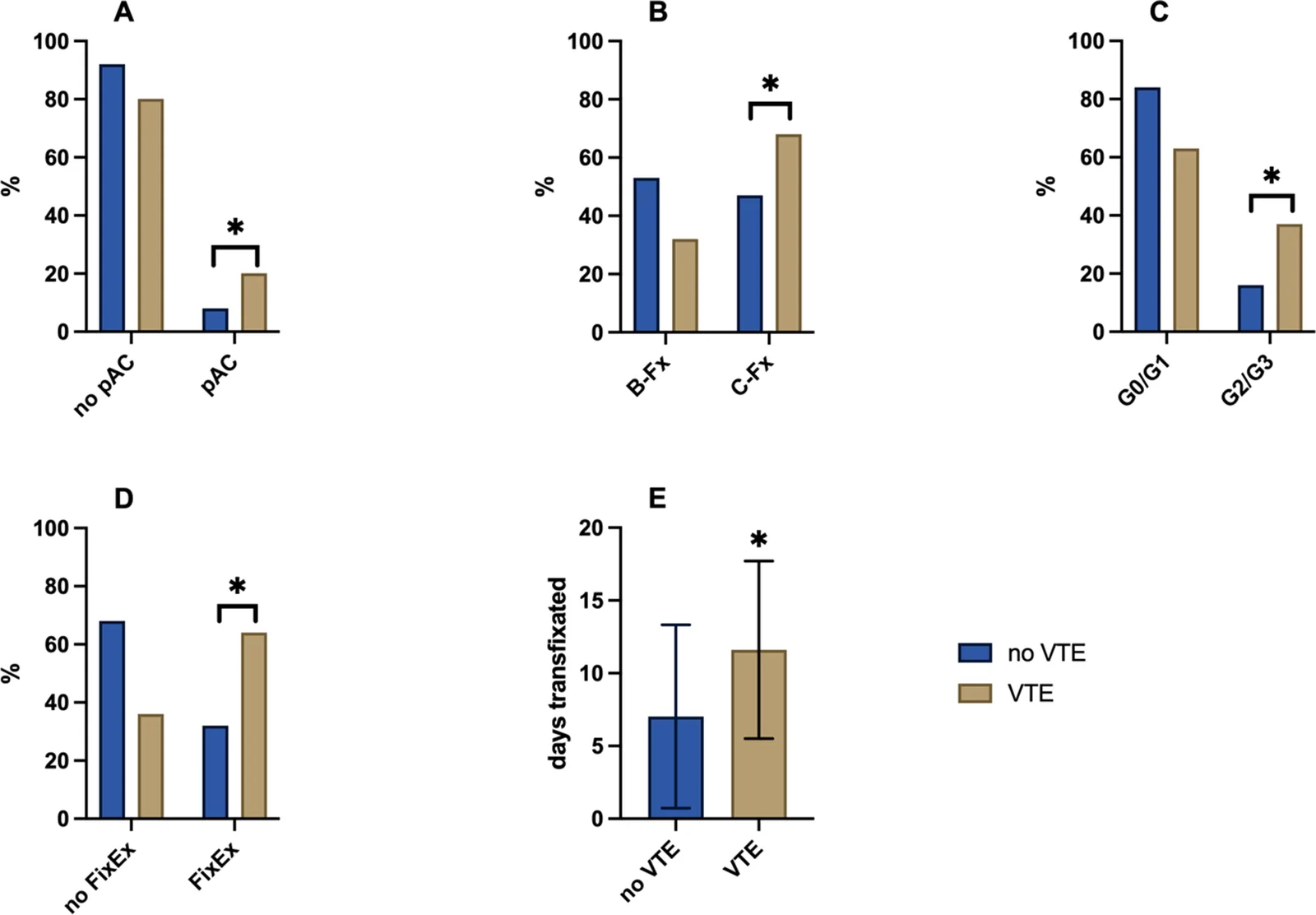
Distribution of patient- and injury-related risk factors stratified by VTE occurrence. Bar charts show the percentage of patients with and without VTE based on patient- and injury-related variables. Values are reported as mean ± SD or n (%); Asterisks indicate *p* < 0.05 (**A**) pre-existing anticoagulation (pAC) (**B**) fracture type acc. to AO-classification (**C**) degree of closed soft tissue damage acc. to Tscherne and Oestern classification (**D**) initial use of external fixator (**E**) duration of external transfixation

In the preoperatively screened TPF cohort (*n* = 205), preoperative patient- and injury-related risk factors were evaluated, comparing fractures with VTE (DVT + MVT + PE; *n* = 41) with those without VTE (*n* = 164; see Table 1; Fig. 4). All comparisons were performed at fracture level.

**Fig. 5:**
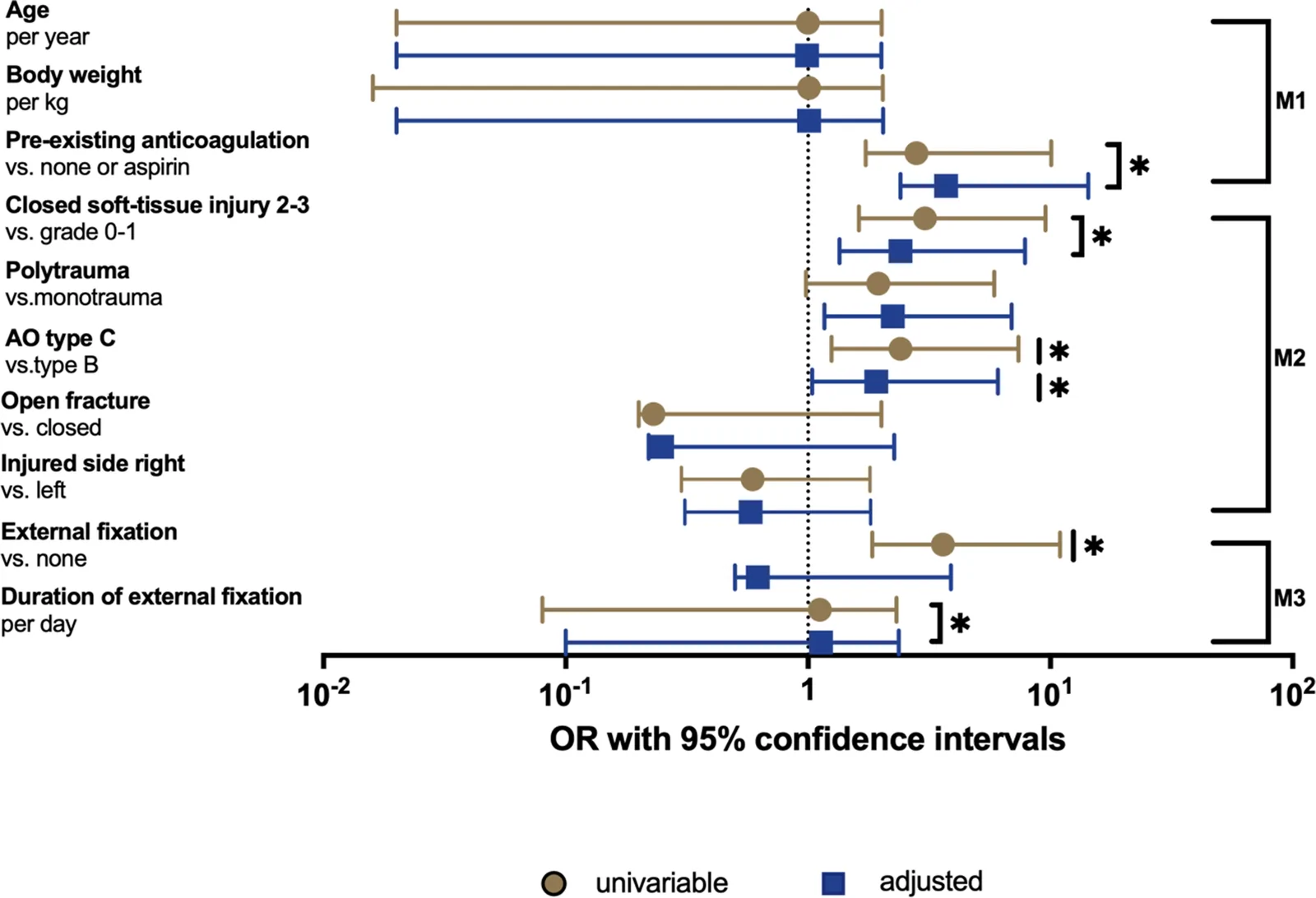
Forest plot of odds ratios (ORs) with 95% confidence intervals (CIs) for factors associated with preoperative VTE in the routinely screened cohort [B], analysed at fracture level (41 events, 205 fractures). Each variable is shown twice: the univariable estimate (tan circles, upper bar) and the corresponding adjusted estimate (blue squares, lower bar). Because only 41 events were available, the covariates were not entered into a single model but grouped into clinically defined models, indicated by the brackets on the right: M1, age, body weight and pre-existing antithrombotic therapy; M2, grade of closed soft-tissue injury, AO classification, injury pattern, open fracture and injured side; M3, use and duration of external fixation. A fourth, severity-adjusted model (grade of closed soft-tissue injury, injury pattern and duration of external fixation) is reported in the text only and is not plotted, so that each variable appears with a single adjusted estimate. ORs are given per unit increase for continuous variables (age per year, body weight per kg, duration of external fixation per day) and against the stated reference category for categorical variables; the corresponding numerical estimates are given in the Results. The x-axis is logarithmic and the dotted vertical line marks the null effect (OR = 1). Asterisks indicate *p* < 0.05

Regarding patient-related factors, pre-existing antithrombotic medication was significantly more frequent among fractures with VTE than among those without (19.5% vs. 8.0%; *p* = 0.030; Table 1). In the patient-related multivariable model, which comprised age, body weight and pre-existing antithrombotic therapy (*n* = 204 patients; three covariates, 41 events, events-per-variable ratio 13.7), pre-existing therapeutic anticoagulation with a direct oral anticoagulant or phenprocoumon (reference category: no anticoagulation or aspirin) was independently associated with VTE (adjusted OR 2.96, 95% CI 1.10–7.98; *p* = 0.032; see Fig. 5).

Because the indication for anticoagulation was not retrievable from the records, this variable may represent a surrogate marker of previous thromboembolic disease or of a higher baseline cardiovascular risk (confounding by indication) rather than a direct risk factor, and it is interpreted accordingly throughout this manuscript. In the same model, neither age (adjusted OR 0.996 per year, 95% CI 0.975–1.017; *p* = 0.691) nor body weight (adjusted OR 1.009 per kg, 95% CI 0.992–1.026; *p* = 0.288) was independently associated with VTE. Model diagnostics indicated no relevant multicollinearity (maximum VIF 1.09) and acceptable discrimination (c-statistic 0.62), although explained variation was low (Nagelkerke R² 0.04) and calibration was imperfect (Hosmer–Lemeshow *p* = 0.020).

With respect to injury characteristics, AO type C fractures were significantly more frequent among fractures with VTE than among those without VTE (68.3% vs. 47.2%; *p* = 0.016). Similarly, closed high-grade soft-tissue injury (grade 2–3 acc. to Tscherne and Oestern) was likewise more common (36.6% vs. 16%; *p* = 0.003). Moreover, VTE was detected more frequently in fractures managed with external fixation (63.4% vs. 32.5%; *p* < 0.001; see Fig. 4).

In the injury-related logistic regression model, which comprised the grade of closed soft-tissue injury, the AO classification, the injury pattern, the presence of an open fracture and the injured side (*n* = 205 complete cases, five covariates, 41 events, events-per-variable ratio 8.2), a high-grade closed soft-tissue injury (grade 2–3 vs. 0–1 acc. to Tscherne and Oestern: OR 2.41, 95% CI 1.07–5.45; *p* = 0.035) and the injury pattern (polytrauma vs. monotrauma: OR 2.24, 95% CI 1.07–4.68; *p* = 0.032) were significantly associated with VTE, whereas the AO classification (type C vs. type B: OR 1.91, 95% CI 0.87–4.17; *p* = 0.106), an open fracture (open vs. closed: OR 0.25, 95% CI 0.03–2.02; *p* = 0.194) and the injured side (right vs. left: OR 0.58, 95% CI 0.27–1.23; *p* = 0.156) were not. Model diagnostics indicated no relevant multicollinearity (maximum VIF 1.12) and moderate discrimination (c-statistic 0.70; Nagelkerke R² 0.13), with adequate calibration (Hosmer–Lemeshow *p* = 0.084). In separate univariable models, both the use of external fixation (OR 3.60, 95% CI 1.76–7.35; *p* < 0.001) and its duration (OR 1.12 per day, 95% CI 1.04–1.20; *p* = 0.004) were significantly associated with VTE (see Fig. 5).

In the multivariable logistic regression model adjusted for injury severity, which comprised the grade of closed soft-tissue injury, the injury pattern and the duration of external fixation (*n* = 204 complete cases, three covariates, 41 events, events-per-variable ratio 13.7), soft-tissue damage (grade 2–3 vs. 0–1: OR 1.42, 95% CI 0.52–3.89; *p* = 0.495) and the injury pattern (polytrauma vs. monotrauma: OR 1.96, 95% CI 0.76–5.05; *p* = 0.166) were no longer significantly associated with VTE, whereas the duration of external fixation remained independently associated with VTE (OR 1.11 per day, 95% CI 1.02–1.20; *p* = 0.011). Model diagnostics were comparable to those reported below for the model combining fixator use and duration (maximum VIF 1.34, c-statistic 0.73, Nagelkerke R² 0.17), again with imperfect calibration (Hosmer–Lemeshow *p* < 0.001); discrimination and effect estimates were nonetheless stable and consistent with the univariable analyses. Given the limited number of events, the width of the confidence intervals indicates limited precision, and the adjusted estimates should be regarded as exploratory. When the use of external fixation and its duration were entered into the same model (*n* = 204 complete cases, two covariates, 41 events, events-per-variable ratio 20.5), the association was carried entirely by the duration (OR 1.13 per day, 95% CI 1.03–1.24; *p* = 0.007), whereas the use of an external fixator itself was no longer associated with VTE (OR 0.62, 95% CI 0.12–3.27; *p* = 0.570). In this combined model, multicollinearity was more pronounced given the correlation between fixator use and its duration (maximum VIF 2.98); discrimination was moderate (c-statistic 0.71; Nagelkerke R² 0.15), but calibration was poor (Hosmer–Lemeshow *p* < 0.001), most likely reflecting the strongly right-skewed distribution of external fixation duration.

Two sensitivity analyses were performed. First, restricting the outcome to DVT and PE only (excluding isolated MVT; 35 events) did not materially change the pattern of associations: pre-existing anticoagulation remained independently associated with the outcome (OR 4.03, 95% CI 1.45–11.21; *p* = 0.007), as did the duration of external fixation (OR 1.08–1.10 per day, *p* ≤ 0.021 across models). Second, repeating the patient-related model at the patient level (*n* = 204, one bilateral case counted once) yielded virtually unchanged estimates (anticoagulation OR 2.93, 95% CI 1.09–7.91; *p* = 0.033), confirming that clustering by patient did not materially affect the results.

Rather than a matched-pair analysis, which would have further reduced the number of events available for adjustment, a receiver operating characteristic (ROC) analysis was performed to identify a data-driven threshold for the duration of external fixation as a predictor of VTE (area under the curve 0.71). The Youden-optimal cut-off was 9 days (sensitivity 53.7%, specificity 86.4%), beyond which the risk of VTE increased markedly; this threshold is provided as a pragmatic reference point rather than a validated clinical decision rule, given the limited number of events.

**Table 1:** Patient-, injury-, and management-related factors in surgically treated tibial plateau fractures with versus without preoperative venous thromboembolism (VTE), shown separately for the symptom-triggered cohort [A] and the routinely screened cohort [B]. Unit of analysis: all comparisons were performed at fracture level. Patient-related characteristics, and the injury characteristics marked †, are attributes of the patient and were assigned to each fracture of the respective patient; the corresponding patient counts are given in the column headings

Factor	Unit / classification	Cohort [A] — symptom-triggered DUS			Cohort [B] —preoperative screening		
		VTE 9 fractures 9 patients	No VTE 237 fractures 234 patients	*p*	VTE 41 fractures 41 patients	No VTE 164 fractures 163 patients	*p*
**Patient-related factors**							
Age	years	61.2 ± 13.7	55.5 ± 15.9	0.318	54.6 ± 17.4	55.0 ± 17.7	0.922
Body mass index	kg/m²	26.9 ± 4.6	26.6 ± 5.1	0.848	28.0 ± 6.9	26.9 ± 5.8	0.362
Height	cm	172.4 ± 11.1	172.1 ± 9.3	0.748	172.2 ± 8.9	171.0 ± 9.9	0.569
Body weight	kg	79.8 ± 12.6	78.7 ± 16.8	0.600	83.5 ± 21.1	79.4 ± 20.0	0.163
Preoperative haemoglobin	mmol/L	7.1 ± 1.1	7.8 ± 1.2	0.074	7.3 ± 1.1	7.6 ± 1.1	0.115
Sex	male / female	4 (44.4) / 5 (55.6)	113 (47.7) / 124 (52.3)	0.999	20 (48.8) / 21 (51.2)	72 (44.2) / 91 (55.8)	0.604
Weight category	normal weight	4 (44.4)	87/234 (37.2)	0.478	12 (29.3)	64 (39.3)	0.415
	overweight	2 (22.2)	97/234 (41.5)		19 (46.3)	59 (36.2)	
	obesity	3 (33.3)	50/234 (21.4)		10 (24.4)	40 (24.5)	
ASA classification	ASA 1–2 / ASA 3–4	4 (44.4) / 5 (55.6)	198 (83.5) / 39 (16.5)	0.011*	34 (82.9) / 7 (17.1)	128 (78.5) / 35 (21.5)	0.533
Pulmonary comorbidity	no / yes	9 (100) / 0	210 (88.6) / 27 (11.4)	–	28/30 (93.3) / 2/30 (6.7)	101/118 (85.6) / 17/118 (14.4)	0.258
Diabetes mellitus	no / yes	9 (100) / 0	218 (92.0) / 19 (8.0)	–	38 (92.7) / 3 (7.3)	150 (92.0) / 13 (8.0)	0.889
Osteoporosis	no / yes	9 (100) / 0	213 (89.9) / 24 (10.1)	–	22/30 (73.3) / 8/30 (26.7)	95/118 (80.5) / 23/118 (19.5)	0.388
Cardiovascular comorbidity	no / yes	5 (55.6) / 4 (44.4)	153 (64.6) / 84 (35.4)	0.725	29 (70.7) / 12 (29.3)	127 (77.9) / 36 (22.1)	0.332
Malignancy	no / yes	9 (100) / 0	233 (98.3) / 4 (1.7)	–	38 (92.7) / 3 (7.3)	157 (96.3) / 6 (3.7)	0.311
Pre-existing antithrombotic medication	none or aspirin / DOAC or phenprocoumon	9 (100) / 0	229 (96.6) / 8 (3.4)	–	33 (80.5) / 8 (19.5)	150 (92.0) / 13 (8.0)	0.030*
Smoking	no / yes	9 (100) / 0	192 (81.0) / 45 (19.0)	–	31 (75.6) / 10 (24.4)	124/161 (77.0) / 37/161 (23.0)	0.849
Alcohol abuse	no / yes	8 (88.9) / 1 (11.1)	214 (90.3) / 23 (9.7)	0.999	38 (92.7) / 3 (7.3)	138/162 (85.2) / 24/162 (14.8)	0.439
**Injury-related factors**							
Injury energy ^†^	low / high energy	2 (22.2) / 7 (77.8)	134/235 (57.0) / 101/235 (43.0)	0.082	26 (63.4) / 15 (36.6)	111/162 (68.5) / 51/162 (31.5)	0.533
Mechanism of force ^†^	direct / indirect	9 (100) / 0	189/235 (80.4) / 46/235 (19.6)	–	31 (75.6) / 10 (24.4)	115/162 (71.0) / 47/162 (29.0)	0.556
Injury pattern ^†^	monotrauma / polytrauma (or similar)	4 (44.4) / 5 (55.6)	161/235 (68.5) / 74/235 (31.5)	0.154	22 (53.7) / 19 (46.3)	113 (69.3) / 50 (30.7)	0.058
Closed soft-tissue injury (Tscherne & Oestern)	grade 0–1 / grade 2–3	5 (55.6) / 4 (44.4)	171 (72.2) / 66 (27.8)	0.279	26 (63.4) / 15 (36.6)	137 (84.0) / 26 (16.0)	0.003*
Fracture type	closed / open	8 (88.9) / 1 (11.1)	233 (98.3) / 4 (1.7)	0.171	40 (97.6) / 1 (2.4)	147 (90.2) / 16 (9.8)	0.127
AO classification	type 41B / type 41 C	2 (22.2) / 7 (77.8)	136 (57.4) / 101 (42.6)	0.045*	13 (31.7) / 28 (68.3)	86 (52.8) / 77 (47.2)	0.016*
Fracture-dislocation (Moore)	no / yes	0 / 9 (100)	111/236 (47.0) / 125/236 (53.0)	–	22 (53.7) / 19 (46.3)	83 (50.9) / 80 (49.1)	0.754
Compartment syndrome	no / yes	8 (88.9) / 1 (11.1)	217 (91.6) / 20 (8.4)	0.558	36 (87.8) / 5 (12.2)	158 (96.9) / 5 (3.1	0.030*
Affected side	left / right	7 (77.8) / 2 (22.2)	137 (57.8) / 100 (42.2)	0.313	27 (65.9) / 14 (34.1)	87 (53.4) / 76 (46.6)	0.150
**Management-related factors**							
External fixation	no / yes	2 (22.2) / 7 (77.8)	158 (66.7) / 79 (33.3)	0.010*	15 (36.6) / 26 (63.4)	110 (67.5) / 53 (32.5)	< 0.001*
Duration of external fixation	days	15.43 ± 13.73	9.90 ± 5.83	0.300	11.55 ± 6.06	7.02 ± 6.30	< 0.001*
Time to definitive surgery	days	10 [8–14]	6 [4–10]	0.009*	13 [11–17]	7 [5–9]	< 0.001*
Length of hospital stay ^†^	days	23 [21–26]	12 [9–17]	< 0.001*	21 [17–29]	12 [8–17]	< 0.001*

Three patients in cohort [A] and one patient in cohort [B] sustained bilateral fractures; none of them developed VTE, so the VTE columns are unaffected. In cohort [B] the two sides of the bilateral patient were recorded as a single observation, so the non-VTE column of this cohort is based on 163 of the 164 fractures throughout; the same applies to weight category in cohort [A] (234 of 237). Missing data: owing to the retrospective design, documentation was not complete for every variable. Values are given as n (%) where the variable was documented in all cases of the respective column and as n/N (%) where documentation was incomplete, N denoting the number of documented cases; percentages always refer to documented cases (valid percent). In cohort [A] documented cases ranged from 234/237 to 237/237 in the patient-related block and from 235/237 to 237/237 in the injury- and management-related blocks; in cohort [B] they ranged from 30/41 (73.2%) to 41/41 in the VTE column and from 118/163 (72.4%) to 163/163 in the non-VTE column. No imputation was performed and no case was excluded from the descriptive comparisons. Pulmonary comorbidity and osteoporosis, the only variables with more than 10% missing data, were reported descriptively and were not entered into any of the multivariable models; all categorical covariates of these models were documented in every case, so the complete-case analysis corresponds to the full analytic sample. For categorical variables both levels are given as n (%). Continuous variables are reported as mean ± standard deviation. Asterisks indicate *p* < 0.05. † patient-level attribute assigned to each fracture of the respective patient. ASA, American Society of Anesthesiologists physical status; AO, AO/OTA classification; DOAC, direct oral anticoagulant; DUS, duplex compression ultrasound; VTE, venous thromboembolism. Time to definitive surgery and length of hospital stay were both markedly longer in fractures with preoperative VTE; given that a detected preoperative VTE itself delays surgery and prolongs hospitalisation in our protocol (see Methods), these associations most likely reflect this reverse-causal pathway rather than these variables acting as risk factors for VTE, and they are reported descriptively for completeness only

## Discussion

Tibial plateau fractures are associated with a relatively high incidence of preoperative DVT. Studies have shown that the incidence of preoperative DVT in tibial plateau fractures ranges from 16.3% to 23.6% [24, 29]. The present data are in line with these findings, with a detected preoperative DVT prevalence of 17.1% (95% CI 12.5–22.8) and a detected overall VTE prevalence of 20.0% (95% CI 15.1–26.0) under routine screening. For example, one study reported that 15.3% of patients with tibial plateau fractures had distal DVT, while only 1% had proximal DVT [24], which is comparable to the present data with most DVTs being distal (83% distal vs. 17% proximal). Other authors found that 31.7% of patients with low-energy tibial plateau fractures and 40.2% of patients with high-energy tibial plateau fractures had preoperative DVT, with no significant difference between the two groups [34]. Our findings are consistent with an association between severe closed soft-tissue damage — and, less consistently, higher AO fracture types — and the detection of thromboembolic events; the retrospective design does not permit conclusions regarding causality.

It can be proposed that initially described prevalence rates in most studies refer to clinically manifest DVTs [14–22]. In a limited number of additional studies, in which systematic ultrasound screening for tibial plateau fracture patients was routinely conducted [16, 26, 29], a markedly increased incidence of DVT has been shown, indicating a high number of unreported cases.

As many studies do not differentiate between clinically manifest and asymptomatic/ inapparent DVTs, it is reasonable to assume that thrombotic incidents are greatly under-recorded and a substantial proportion of asymptomatic and undetected DVTs existed within these investigations. In the screened cohort of the present study, 13.7% of all screened fractures (28/205; 95% CI 9.6–19.0) harboured a clinically inapparent (“silent”) DVT or MVT, corresponding to 68% of all detected events, whereas only 6.3% of screened fractures (13/205; 95% CI 3.7–10.5) exhibited signs and symptoms suspicious for DVT/MVT. In addition, timely and correct diagnosis of DVT/MVT could be comparably difficult as differentiation between fracture symptoms and venous thrombosis symptoms may be impeded in the acute setting. The present data also show a noteworthy disparity between VTE diagnoses made on the basis of clinical symptoms and suspicion compared to those detected through routine DUS screening. Consequently, in the absence of routine screening, a substantial number of thromboembolic events remain undetected and undiagnosed, leaving physicians unaware of the patient’s thrombotic risk and thus unable to initiate therapeutic measures to prevent progression of DVT/MVT or potentially fatal PE.

In one patient of the unscreened cohort the fatal pulmonary embolism originated from a deep vein thrombosis that had not been recognised preoperatively because screening had not yet been introduced. Whether routine screening would have prevented this death cannot be established from a single retrospective case. Notably, the second in-hospital cardiac arrest occurred in a patient whose proximal DVT had been detected by screening and treated with therapeutic anticoagulation, which underlines that detection alone does not eliminate the perioperative thromboembolic risk.

The present study compared two diagnostic strategies and was not designed to determine whether routine preoperative screening improves patient-relevant outcomes. The number of detected preoperative pulmonary embolisms was low in both cohorts and the difference between them was not statistically significant, and no data on post-thrombotic syndrome, functional outcome or bleeding complications arising from screening-triggered anticoagulation were available. Routine screening also detects many distal and isolated calf muscle vein thromboses whose clinical significance remains debated and whose treatment carries a bleeding risk [35]. Whether the substantially higher diagnostic yield translates into a net clinical benefit therefore remains unresolved and should be addressed prospectively.

Although the exact degree to which the presence and the extent (proximal/distal) of a DVT affects clinical outcome following TPF remains unclear. Apart from the undoubted fact that the risk for the fearful and fatal complication of PE is markedly increased, a significant delay of the definitive surgical treatment complicates and reduces the quality of anatomic articular reduction and fixation, thereby adversely affecting functional outcome and the risk of posttraumatic osteoarthritis. Additionally, prolonged hospitalization and costs as well as postthrombotic syndrome with pain, edema, skin changes are frequent and often irreversible consequences.

To date, only few studies with very different design have tried to identify risk factors that contribute to the incidence of DVT in TPF patients, which, however, revealed partly heterogeneous, inconsistent and also controversial results [24–26, 29, 36]. Since detailed understanding of these risk factors holds the key for effective risk stratification and timely targeted prevention strategies the present study collectively analyzes for the first time very detailed the patient- and injury related risk factors for VTE in TPF patients.

### Patient-related risk factors

Advancing age is consistently described as a significant risk factor for VTE. Patients aged 41 years and above are expected to have a higher risk compared with younger individuals [26, 36]. Similarly, male sex seems also frequently to be associated with an increased risk of VTE in TPF patients [24, 25, 27]. In addition, common comorbidities, e.g. diabetes and particularly hypertension are known to represent relevant risk factors for VTE in several studies [24, 26, 37]. Interestingly, elevated alkaline phosphatase and D-dimer levels are reported to be linked with increased DVT risk, although D-dimer levels can also be heightened in response to the initial trauma and/or surgical intervention [24, 26, 38].

In the present study, higher body weight was not statistically different between groups and not independently associated with thrombotic events in the multivariable model. In the present cohort, neither sex, age, nor body weight/BMI was significantly associated with VTE; given the limited number of events, however, a clinically relevant association of these variables cannot be excluded.

Pre-existing antithrombotic medication was significantly associated with the detection of VTE (OR 2.96, 95% CI 1.10–7.98). This must not be read as a pharmacological effect. The indication for anticoagulation was not retrievable from the records, and patients on long-term anticoagulation are by definition selected for previous thromboembolic disease, atrial fibrillation or a higher baseline cardiovascular risk, so that confounding by indication is the most likely interpretation.

A second, not mutually exclusive explanation is the perioperative interruption and dose reduction of anticoagulation required to permit surgery, during which patients are transiently under-anticoagulated relative to their baseline requirement. The present data cannot distinguish between these explanations.

### Injury-related risk factors

In previous studies, high-energy trauma, particularly open fractures have been reported to be associated with a higher incidence of DVT, most likely caused by trauma-induced tissue damage and consecutive proinflammatory response [24, 27]. Notably, our findings showed a significant effect of closed soft-tissue damage on the incidence of VTEs while this significant result could not be confirmed for different grade of open TPF. Among the different causative factors, the relatively small sample size of open TPFs in the preoperatively screened cohort (*n* = 17), providing insufficient statistical power for analysis, may be the main reason.

However, particularly closed soft tissue damage is known to be associated with tremendous secondary and prolonged microvascular perfusion failure, inflammatory response as well as reduced muscle performance and pump, known to synergistically trigger the development of VTE risk [12, 39]. Consequently, especially patients with a high-grade soft-tissue damage should be perioperatively monitored for the development of DVT and routinely receive routine DUS screening.

Additionally, the initial use of an external fixator was associated with the detection of preoperative VTE in univariable analysis (OR 3.60, 95% CI 1.76–7.35; *p* < 0.001). External fixation is not randomly allocated but is used preferentially in type C fractures, in polytrauma and in substantial soft-tissue damage, and therefore acts at least in part as a surrogate marker of injury severity (confounding by indication). Importantly, this association did not persist once the duration of external fixation was taken into account: in a model containing both variables only the duration remained associated with VTE (OR 1.13 per day, 95% CI 1.03–1.24; *p* = 0.007), whereas the use of a fixator itself was not (OR 0.62, 95% CI 0.12–3.27; *p* = 0.570). Since use and duration are structurally linked this model must be read with caution, but it indicates that the association is driven by the length of the period spent in the fixator rather than by the application of a fixator as such.

The duration of external fixation remained associated with VTE after adjustment for injury severity (OR 1.11 per day, 95% CI 1.02–1.20; *p* = 0.011), corresponding to approximately 11% higher odds per additional day. Prolonged immobilisation with venous stasis due to an impaired muscle pump is a plausible but unproven explanation. However, the duration is itself determined by the soft-tissue condition and hence by injury severity, and reverse causation is also conceivable, since a detected thrombosis delayed definitive fixation in our protocol and thereby prolonged the time spent in the fixator. These observations are consistent with performing definitive fixation as soon as the soft-tissue condition allows, but they do not demonstrate that shortening external fixation reduces VTE. Our findings are in line with Hermel et al. [29], who also reported high-energy trauma, severe injury of the tibial plateau (in particular compartment syndrome) and the use of an external fixator as factors associated with preoperative DVT.

Furthermore, the present data are compatible with the notion that trauma severity and soft-tissue damage contribute to an increased VTE risk, as a statistically significant association was observed between higher AO classification grades and VTE incidence (B-type vs. C-type fractures). However, this effect must be interpreted with caution since it could not be reproduced as an independent risk factor in the multivariable analysis.

### Management of patients with preoperative VTE

When planning definitive fixation, the choice of single or combined surgical approaches primarily depends on fracture type and the extent of soft tissue damage. In patients with preoperative DVT, the decision to employ posterior surgical approaches should be made with caution. Conversely, patients treated via posterior approaches should be closely monitored postoperatively for the development of VTE, and routine postoperative DUS should be performed. Furthermore, the use of a tourniquet should be avoided, as it may promote VTE development [40]. In our own practice, tourniquet use was predominantly avoided whenever the operative field permitted.

In our practice the detection of a preoperative thrombosis modified the timing of surgery and the perioperative medical management far more often than the surgical approach itself, which is dictated by fracture morphology: a posteromedial or posterolateral fragment cannot be reduced and buttressed from an anterior window. A popliteal or more proximal DVT therefore did not lead us to abandon a necessary posterior approach, but to postpone surgery until re-imaging confirmed a stable or regressing thrombus under therapeutic anticoagulation, to prefer a posteromedial rather than a direct posterior window whenever the fragment allowed it, and to intensify intraoperative monitoring for pulmonary embolism. In isolated distal DVT or in MVT the approach was not modified.

Once a preoperative DVT had been diagnosed, management followed the algorithm shown in Fig. 2, and the principal clinical consequence was a delay of definitive fixation.

The use of inferior vena cava filters (IVCF) remains a subject of considerable debate. Current indications include patients with acute DVT and/or concomitant PE who have absolute contraindications to therapeutic anticoagulation, who have failed anticoagulation therapy, or in whom anticoagulation cannot be safely maintained due to the risk of perioperative hemorrhage. At the current state, the use as a prophylaxis in high-risk orthopedic procedures are not supported [41–46]. While the use of an IVCF in patients with distal DVT is usually not indicated, the application of an adjuvant IVCF in high-risk patients with proximal DVT, failed anticoagulation therapy and timely surgical therapy with potential hemorrhage, might be appropriate [46]. Despite inherent procedural risks (such as filter thrombosis and dislocation) [44, 45], there appears to be a lack of alternative solutions. Therefore, the use of a IVCF in patients with TPFs may warrant further investigation as it has been reported to be potentially effective in preventing PE in TPF patients, due to a more generous use also in high-risk patients without failed anticoagulation therapy but proximal DVT and the need for posterior approaches [29, 47].

That injury severity and soft-tissue damage are associated with preoperative VTE is not unexpected; its practical value lies in the fact that these variables are available at admission. In the present cohort, high-grade closed soft-tissue injury (Tscherne–Oestern grade 2–3), primary external fixation and pre-existing antithrombotic medication identified fractures with a markedly higher detection rate of VTE. Where routine screening of every patient is not feasible, preoperative DUS should therefore be mandatory in patients with at least one of these features and in all patients whose definitive fixation is expected to be delayed.

Acute compartment syndrome combines severe soft-tissue injury, prolonged immobilisation and the additional surgical trauma of fasciotomy, and was identified as a risk factor for preoperative DVT by Hermel et al. [29]. We therefore regard patients undergoing fasciotomy as a high-risk group who should be screened before definitive fixation irrespective of symptoms.

Early definitive fixation and early mobilisation are central to the treatment of TPF, and both are relevant to thromboembolic risk. Fractures not stabilised with an external fixator were immobilised preoperatively in a knee immobiliser. Postoperatively, the permitted range of motion was defined by the segments of the tibial plateau involved. Partial weight-bearing was prescribed for six weeks. Restoring the calf muscle pump as early as the soft tissues permit is the physiological rationale for keeping the period of external fixation as short as possible.

On the basis of these considerations we propose the following pragmatic pathway, acknowledging that it is derived from retrospective single-centre data and has not been validated prospectively: (i) LMWH prophylaxis combined with mechanical prophylaxis in every patient from admission; (ii) preoperative DUS before definitive fixation in every patient or, where this is not feasible, in the high-risk groups defined above; (iii) surgery as planned if DVT is excluded, intensified anticoagulation and surgery under precaution in MVT, and therapeutic anticoagulation with re-imaging after 7 days in confirmed DVT; (iv) interdisciplinary decision-making with angiology in progressive DVT or PE, including the selective use of an inferior vena cava filter; and (v) postoperative DUS on clinical suspicion and, in our current practice, routinely before discharge after a preoperatively detected thrombosis.

### Limitations

This retrospective study has several limitations. Most importantly, the two cohorts were investigated with different diagnostic strategies: imaging was symptom-triggered in cohort [A] and protocol-based in cohort [B]. The difference in detected prevalence is therefore predominantly attributable to surveillance (ascertainment) bias, which must be regarded as the major limitation of this study. The true prevalence in cohort [A] is unknown and almost certainly underestimated, and every between-cohort comparison has to be read as a comparison of two diagnostic strategies rather than of two populations with a different underlying risk. As the cohorts also represent consecutive time periods, secular changes in patient characteristics, prophylaxis and documentation quality cannot be separated from the effect of screening. A second source of bias is that screening within cohort [B] was itself incomplete, as only 205 of 308 fractures (66.6%) underwent preoperative DUS. The two reasons for this were not random with respect to injury severity: fractures from the introduction period were left unscreened irrespective of severity, whereas the later exceptions were patients admitted from outpatient management and therefore typically less severely injured. The screened population is thus likely to be enriched for more severely injured limbs, so that the reported prevalence may overestimate the prevalence among all fractures treated after 08/2020 and should be read as an estimate for the screened population rather than for cohort [B] as a whole. In addition, the number of events was limited relative to the number of predictors in some models, particularly the combined external fixation use/duration model (events-per-variable ratio 20.5, but with pronounced collinearity between the two covariates); the corresponding estimates should therefore be interpreted with caution and regarded as exploratory rather than confirmatory. Reliance on previously recorded data introduces information bias, and postoperative DUS was performed only on clinical suspicion, so that the rate of asymptomatic postoperative thrombosis cannot be reported. For the same reason, asymptomatic pulmonary embolism cannot be quantified, as routine postoperative CT pulmonary angiography is not justifiable given the associated radiation exposure.

## Conclusion

The findings indicate a substantial burden of preoperative venous thromboembolism in surgically treated TPF. Under routine duplex ultrasound screening the detected VTE prevalence was 16.3% points higher than under symptom-triggered imaging. In this cohort, the degree of closed soft-tissue damage, pre-existing therapeutic anticoagulation and, above all, the duration of external fixation were associated with the detection of preoperative VTE. Only the duration of external fixation is potentially modifiable by the treating surgeon, and it appears reasonable, although unproven, that measures allowing earlier definitive osteosynthesis may also reduce thromboembolic risk. Whether routine screening, risk-adapted thromboprophylaxis or a shorter duration of external fixation improve patient-relevant outcomes must be determined prospectively. Future research should be directed toward specialized anticoagulation protocols and modern preventive strategies, such as pneumatic compression devices and/or vena cava filters.

## Data Availability

The data that support the findings of this study are available from the corresponding author upon reasonable request.
